# Small extracellular vesicles derived from Nrf2-overexpressing human amniotic mesenchymal stem cells protect against lipopolysaccharide-induced acute lung injury by inhibiting NLRP3

**DOI:** 10.1186/s13062-022-00351-9

**Published:** 2022-11-29

**Authors:** Lijuan Xu, Yunlou Zhu, Congye Li, Qixing Wang, Lijie Ma, Junjie Wang, Shouqin Zhang

**Affiliations:** Department of Critical Care Medicine, Shanghai Tenth People’s Hospital, Tongji University School of Medicine, 7th Floor, Building 1, No. 301 Middle Yanchang Road, Jing’an District, Shanghai, 200072 China

**Keywords:** Acute lung injury, Small extracellular vesicles, Nrf2, hAMSCs, NLRP3, Macrophages

## Abstract

**Background:**

Acute lung injury (ALI) is a major cause of respiratory failure in critically ill patients that results in significant morbidity and mortality. Recent studies indicate that cell-based therapies may be beneficial in the treatment of ALI. We recently demonstrated that Nrf2-overexpressing human amniotic mesenchymal stem cells (hAMSCs) reduce lung injury, fibrosis and inflammation in lipopolysaccharide (LPS)-challenged mice. Here we tested whether small extracellular vesicles (sEVs) derived from Nrf2-overexpressing hAMSCs (Nrf2-sEVs) could protect against ALI. sEVs were isolated from hAMSCs that overexpressed (Nrf2-sEVs) or silenced (siNrf2-sEVs) Nrf2. We examined the effects of sEVs treatment on lung inflammation in a mouse model of ALI, where LPS was administered intratracheally to mice, and lung tissues and bronchoalveolar lavage fluid (BALF) were analyzed 24 h later.

**Methods:**

Histological analysis, immunofluorescence microscopy, western blotting, RT-PCR and ELISA were used to measure the inflammatory response in the lungs and BALF.

**Results:**

We found that sEVs from hAMSCs are protective in ALI and that Nrf2 overexpression promotes protection against lung disease. Nrf2-sEVs significantly reduced lung injury in LPS-challenged mice, which was associated with decreased apoptosis, reduced infiltration of neutrophils and macrophages, and inhibition of pro-inflammatory cytokine expression. We further show that Nrf2-sEVs act by inhibiting the activation of the NLRP3 inflammasome and promoting the polarization of M2 macrophages.

**Conclusion:**

Our data show that overexpression of Nrf2 protects against LPS-induced lung injury, and indicate that a novel therapeutic strategy using Nrf2-sEVs may be beneficial against ALI.

## Background

Acute lung injury (ALI) is a major cause of acute respiratory failure resulting in significant morbidity and mortality in critically ill patients [1, 2]. ALI is characterized by widespread inflammation of the lungs, and accumulation of inflammatory macrophages and neutrophils that result in damage to the pulmonary endothelial cells and epithelial barriers [3]. Patients with sepsis or pneumonia are highly susceptible to ALI, demonstrating a link to infection [4]. Hospital acquired pneumonia (HAP) is mostly caused by Gram-negative bacilli, including Streptococcus pneumoniae, Pseudomonas aeruginosa, Haemophilus influenzae and Escherichia coli, all of which are aerobic bacteria [5]. Lipopolysaccharide (LPS) is a constituent of the outer membrane of the cell wall of Gram-negative bacteria, and is widely used to induce ALI in animal models due to its high reproducibility, good sensitivity and low cost [6]. Current therapies for ALI include mechanical ventilation and immunosuppression, and although advances in ALI-targeted therapies have been made, the patient survival rate remains low [7]. Thus, the development of novel therapeutic strategies is required to improve the survival rate of ALI patients.

Several studies have indicated that cell-based therapies such as the use of mesenchymal stem cells (MSCs) are potentially beneficial in the treatment of ALI [3, 8]. MSCs are multi-potent stem cells that have the ability to differentiate into osteoblasts, adipocytes and chondrocytes [9]. Transplantation of MSCs isolated from bone marrow has been shown to ameliorate inflammation in multiple models of inflammatory disease including colitis [10] and ALI [11–14]. As access to bone marrow MSCs is difficult, additional sources of MSCs have been examined. MSCs from the human amniotic membrane (hAMSCs) are more easily obtained and have been shown to be beneficial in a wide range of diseases including myocardial infarction, intracerebral hemorrhage, spinal cord injury and Alzheimer’s disease [15–18]. We have previously demonstrated that hAMSCs protect against LPS-induced lung inflammation [19]. However, MSCs are complex to produce and transport, and transplanted MSCs less likely to survive in an inflamed microenvironment [20]. Thus, the use of cell-free small Extracellular Vehicles (sEVs) derived from MSCs may limit inflammatory disease while reducing side effects [21].

The most common vectors for gene transport are viruses and plasmids, which are highly virulent and rarely specific [22]. sEVs with 50–150 nm diameter that have been shown to contain proteins, DNA, RNA, lipids and various metabolites that have potential inflammatory and immunomodulatory functions [23, 24]. Genetic modification at the cellular level can make the sEVs secreted by modified cells being targeted, so as to achieve the purpose of efficient and safe transport of protein or RNA molecules [25]. Therefore, sEVs can be used as vectors for gene therapy. Recent studies point to a therapeutic role for sEVs in ALI [26–30]. However, whether sEVs derived from hAMSCs protect against ALI remains unknown.

Nuclear factor (erythroid-derived 2)-like 2 (Nrf2) is a transcription factor involved in regulating antioxidant processes including the Nrf2-antioxidant response element pathway, which functions as a major cell defense mechanism against oxidative damage caused by injury and inflammation [31]. In recent years, Nrf2 has emerged as a promising target for the treatment of multiple diseases [32] including cancer [33], cardiocerebral vascular disease [34], neurodegenerative diseases [35] and respiratory diseases[36]. In a model of LPS-induced lung injury, Nrf2 activation was shown to protect against disease development [37]. Furthermore, we demonstrated that overexpression of Nrf2 enhanced the efficacy of hAMSCs to limit LPS-induced lung injury [19]. Thus, modulation of Nrf2 expression may provide a novel approach to treat ALI.

Nrf2 has been shown to regulate expression of many downstream genes [38] including the NLRP3 inflammasome [39]. A recent study demonstrated that the NLRP3 inflammasome has a role in inducing apoptosis in the lung via a ROS/Nrf2/NLRP3-mediated pathway [40], and several studies have suggested that Nrf2 has the potential to be used as a therapeutic target to inhibit the assembly of the NLRP3 inflammasome complex [32]. Further, it has been shown that activation of the NLRP3 inflammasome is critical for the development of LPS-induced lung inflammation in mice [41, 42]. Thus, the Nrf2/NLRP3 axis could be a potential therapeutic target to treat ALI.

In the present study, we show that sEVs generated from hAMSCs are protective in a mouse model of LPS-induced lung inflammation. sEVs derived from Nrf2-overexpressing hAMSCs (Nrf2-sEVs) further enhanced protection, while knockdown of Nrf2 inhibited the protective effects. Mechanistically, treatment with Nrf2-sEVs inhibited activation of the NLRP3 inflammasome, reduced production of inflammatory cytokines and promoted the polarization of M2 macrophages. Thus, Nrf2-sEVs are a viable therapeutic strategy to treat ALI.

## Materials and methods

### Animals

Male C57BL/6J mice (8–12 weeks old, weighing 20–25 g) were purchased from Shanghai Jiesijie Laboratory Animal Co., LTD (Shanghai, China). The care and use of the animals conformed to the Guide for the Care and Use of Laboratory Animals. Mouse study protocols were approved by the Institutional Animal Care and Use Committee of Shanghai Tenth People’s Hospital, and the approval number was SHDSYY-2019-2363.

### hAMSC cell culture and transduction

hAMSCs (CRL-4035TM) were purchased from ATCC (Manassas, VA, USA), and cultured in Endothelial Cell Basal Medium­2 (EBM­2) (Lonza, Walkersville, MD, USA) supplemented with 10% fetal bovine serum (FBS, Hyclone, Logan, UT, USA) and 1% streptomycin/penicillin at 37 °C, in a humidified atmosphere of 5% CO_2_. Cells at passage 5 were used for all experiments.

hAMSC transduction was performed as described previously [19, 43]. The full-length coding sequence of murine Nrf2 or the following siRNA sequences (siRNA targeting Nrf2 (siNrf2; GCCTTACTCTCCCAGTGAATA) and scramble nonsense siRNA (scramble control; CAACGACTGAGGTATGGTATT)) were purchased from Hanheng Biotechnology (Hanheng Biotechnology Co., Ltd., Shanghai, China) and used to generate lentiviral vectors. To produce lentiviral vectors, 293T cells were transfected with either a lentivirus empty vector or lentivirus vector encoding Nrf2, siNrf2 or control scramble siRNA using Lipofectamine 2000 (Invitrogen, Carlsbad, CA, USA). Supernatants were harvested 72 h later. After centrifugation at 4000 × g for 10 min at 4 °C, supernatants were filtered through a 0.45 μm filter, then centrifuged at 72,000 × g for 120 min at 4 °C. The resulting pellet was resuspended in 500 µl phosphate buffer saline (PBS). hAMSCs were infected with the viral supernatants for 48 h with multiplicity of infection (MOI) of 25. Western blot analysis was used to confirm the transduction efficiencies of the lentiviral vectors.

### Purification and identification of sEVs

sEVs were isolated from the cultural supernatants of hAMSCs by differential centrifugation as described previously [44]. Con-sEVs were derived from empty lentivirus infected hAMSCs, Nrf2-sEVs were derived from hAMSCs transduced with lentivirus overexpressing Nrf2, and siNrf2-sEVs were derived from hAMSCs transduced with lentivirus expressing siNrf2. Briefly, the supernatant of the infected cells was collected 48 h after transfection. The supernatant was centrifuged at 300 × g for 10 min at 4 °C, followed by centrifugation at 10,000 × g for 30 min at 4 °C, and then further centrifugation at 100,000 × g for 120 min at 4 °C. Pellets were resuspended in PBS and centrifuged a final time at 100,000 × g for 120 min at 4 °C. Finally, the pellets were resuspended in PBS (1 mL). Part of the solution (200 µl) was frozen and stored at − 80 °C for subsequent experiments.

The high content of tetraspanins CD9, CD63, and CD81 mean that they are frequently used as identification markers for sEVs [45]. Thus, isolated sEVs were identified by western blotting using antibodies against CD9, CD63, and CD81 (Abcam, UK).

The diameter of sEVs is generally between 30 and 150 nm. Under transmission electron microscopy, sEVs appear round or oval, and are generally bilayer structures with concave sides. The size range and concentration of sEVs were measured by nanoparticle tracking analysis (NTA) with a Nanosight NS 300 system (NanoSight Technology, Malvern, UK) as described previously [46].

The morphology of the sEVs was determined by transmission electron microscopy. Briefly, sEVs were resuspended in PBS. The suspension (20 µl) was dropped onto a copper net with a diameter of 2 nm and left for 2 min at room temperature. sEVs were stained with 3% phosphotungstate solution for 5 min at room temperature. Dried samples were visualized by transmission electron microscopy (Libra 120; Zeiss, Oberkochen, Germany).

### LPS-induced acute lung injury models

ALI models were generated as previously reported [19]. Briefly, mice were anesthetized with intraperitoneal injection of pentobarbital (50 mg/kg). A single dose of LPS (100 µg, Sigma-Aldrich, St. Louis, MO, USA) in 50 µL sterile normal saline (NS) was administered by intratracheal (i.t.) instillation. The mice were then allowed to recover in a 100% oxygen chamber until fully awake. The sham control mice received 0.9% NS instead of LPS. 200 µl purified sEVs (~ 4.5 × 10^9^ particles/ml) were transplanted via the tail vein at 2 and 12 h post-LPS treatment. This experiment was divided into five groups (n = 5 per group): (1) sham, (2) LPS + PBS, (3) LPS + Con-sEVs, (4) LPS + Nrf2-sEVs and (5) LPS + siNrf2-sEVs.

### Lung histology and lung injury score

The left upper lung lobe of each mouse was harvested from mice at 24 h following LPS challenge and fixed in formalin for pathology examination and TUNEL analysis. Briefly, mice were euthanized by cervical dislocation, the alveolar cavity was inflated with 4% buffered formalin for 24 h, and the lobes were dehydrated with gradient alcohol, embedded in paraffin, and cut into 4–5 μm slices. Hematoxylin and eosin (H&E) staining was performed on the lung tissue slices using a H&E Staining Kit according to the manufacturer’s instructions (Servicebio Technology, Wuhan, China). Three different pathologists evaluated and scored the samples in a double-blinded manner. Inflammatory cells in the alveolar airspaces, alveolar walls, the interstitium, hyaline membranes, thickened alveolar septal, and proteinaceous debris filling the airspaces were evaluated to obtain the pulmonary injury score [47]. The final injury score was a successive value ranging from zero to one.

### Measurement of the lung wet to dry weight ratio and arterial oxygen tension (PaO_2_)

The amount of tissue edema was evaluated by calculating the wet to dry weight ratio. Briefly, the left lower lung lobe of each mouse was removed at 24 h following LPS challenge and weighed to obtain the wet weight. The dry weight was measured by drying the lung in an oven at 80 °C for 48 h. A value for the wet to dry weight ratio was calculated by dividing the wet weight by the dry weight.

To evaluate levels of PaO_2_, arterial blood samples from mice were collected at 24 h following LPS challenge. A blood gas analyzer (GEM premier 3000, Instrumentation Laboratory, Bedford, MA, USA) was used to measure PaO_2_.

### TUNEL staining

The paraffin-embedded left upper lung lobe sections were utilized for TUNEL assays in accordance with the manufacturer’s instructions (Roche, Mannheim, Germany). 3% H_2_O_2_ was used to inactivate endogenous peroxidases following deparaffinization and rehydration. Proteinase K was used to permeabilize the nuclei for 20 min at room temperature. Afterwards, endonucleotidyl transferase was added to the mixture and incubated at 37 °C for 1 h. Following the addition of peroxidase-conjugated antibody, color development was conducted using a DAB substrate kit (Sigma). Positive cells were counted using a microscope (Olympus, Tokyo, Japan). Cells that exhibit TUNEL positivity exhibit brown staining in the nucleus, and their results are expressed as a percentage of the total number of cells.

### Measurement of total protein content, inflammatory cell count and cytokine levels in bronchoalveolar lavage fluid (BALF)

Mice were treated with LPS for 24 h, then euthanized by cervical dislocation. BALF (1.5 mL) was collected in PBS containing 0.1 mM EDTA. Samples were centrifuged at 1200 × g for 10 min. The total protein concentration of BALF was measured using a bicinchoninic acid (BCA) protein assay kit according to the manufacturer’s instructions (Beyotime, Shanghai, China). Inflammatory cells were stained using a Wright-Gimsa staining kit for 20 min at room temperature (Jiancheng Company, Nanjing, China) and observed by light microscopy. Levels of pro-inflammatory cytokines (tumor necrosis factor-alpha (TNF-α), interleukin (IL)-6 and IL-1β) in the BALF were analyzed using mouse uncoated ELISA kits (Invitrogen) according to the manufacturer’s instructions. Briefly, samples (100 µl) were added to 96-well ELISA plates and incubated at room temperature for 2 h and plates were read at 450 nm. Each analyte was detected at the following limits: mouse IL-6 at 4 pg/mL, mouse IL-1 at 8 pg/mL, and mouse TNF at 8 pg/mL.

### Real-time quantitative PCR

Total RNA was extracted from the right upper lung lobes using an RNA isolation kit (Roche) and reverse-transcribed at 37 °C for 15 min using a commercial kit from Takara. Expression levels of target genes were determined using the SYBR Premix Ex TaqTM II (Takara, Shiga, Japan) with an ABI 7500 real-time PCR system (Applied Biosystems Inc., Carlsbad, CA, USA). Primers used for reverse transcription and PCR are listed in Table 1. Expression levels were calculated using the ΔΔCt method relative to levels of GAPDH.

**Table 1:** Primers used in the study

Gene	Forward primer 5′→3′	Reverse primer 5′→3′
IL-6	CACGGCCTTCCCTACTTCAC	CCAGTTTGGTAGCATCCATCA
TNF-α	GAGGCACTCCCCCAAAAGAT	TTGCTACGACGTGGGCTAC
iNOS	CAGATCGAGCCCTGGAAGAC	CAACCTTGGTGTTGAAGGCG
CD206	GCTGGCGAGCATCAAGAGTA	CATGCCAGGGTCACCTTTCA
Fizz1	TCCCTCCACTGTAACGAAGA	GGTCCAGTCAACGAGTAAGCA
Arg1	AAGCTTCAGAGAAGTGGCCC	CTGGTGGTGGGTATCACAGG
GAPDH	CACTTGAAGGGTGGAGCCAA	TGATGGCATGGACTGTGGTC

### Western blot analysis

Proteins were analyzed by western blot as previously described [40]. Protein was extracted from the right lower lung lobe tissues using cold RIPA lysis buffer (Santa Cruz Biotechnology, Dallas, TX, USA) and centrifuged at 13,000 × g at 4 °C for 15 min. The protein concentration was determined using a BCA assay kit. 50 µg of protein sample was separated by 10% and 15% SDS-PAGE, and transferred to nitrocellulose membranes (Thermo Fisher Scientific, Waltham, MA, USA).

After transfer, the membrane was blocked with 20 mM Tris-HCl, pH 7.5, 500 mM NaCl, and 0.05% Tween-20 (TBST) (v/v) containing 5% bovine serum albumin (BSA) for 1 h at room temperature and incubated with the following primary antibodies: anti-Nrf2 (1:1000), anti-Lamin B (1:1000), anti-HO-1 (1:2000), anti-NLRP3 (1:1000), anti-IL-18 (1:2000), anti-cleaved Caspase-1 (1:100), anti-ASC (1:2000) and anti-β-actin (1:5000). All the antibodies were obtained from Abcam (Abcam, Cambridge, U.K.). After washing with TBST three times, membranes were incubated with appropriate secondary antibodies for 1.5 h. Finally, the membrane was washed three times with TBST and protein bands were visualized using enhanced chemiluminescence (Thermo Fisher Scientific). The relative expression of protein was calculated as the ratio between the target protein and β-actin or Lamin B.

### Immunofluorescence and immunohistochemistry

Paraffin-embedded left upper lung lobe tissue sections were dewaxed with dimethyl-benzene and hydrated in a graded alcohol series. Then, antigens were retrieved under high pressure. For immunofluorescence staining, the sections were incubated with rat-anti-F4/80 (1:100, Abcam) and rabbit-anti-NLRP3 (1:100, Invitrogen) diluted in 1% donkey serum at 4 °C overnight. After washing the sections three times with PBST, sections were incubated with appropriate secondary antibodies diluted in PBST for 1 h at room temperature. Sections were washed three times as above, and nuclei were stained with DAPI (Invitrogen) for 10 min at room temperature. Finally, the sections were sealed with anti-fluorescence quenching sealant. A confocal microscope (Olympus) was used to examine the sections. The mean optical density (MOD) was analyzed by Image-ProPlus 6.0 software (Media Cybernetics, Inc., Rockville, MD, USA). For immunohistochemistry staining, after overnight incubation at 4 °C with primary antibodies against IL-6 (1:200, Affinity Biosciences, China), TNF-α (1:200, Affinity Biosciences), iNOS (1:200, Affinity Biosciences), CD206 (1:100, Affinity Biosciences), Fizz1 (1:100, Abcam), or Arg1 (1:200, Affinity Biosciences), and then incubated for 45 min with a goat anti-rabbit secondary antibody. As a final step, the sections were stained with hematoxylin and DAB solution, imaged with an Olympus light microscope, and analyzed with ImageJ.

### Statistical analyses

All results were confirmed in at least three independent experiments using the same samples. All quantitative data are presented as mean SD. Statistical analyses were performed using SPSS 21.0 (SPSS Inc., Chicago, IL). ANOVA with the post hoc Tukey-Kramer multiple comparison test was used for comparisons between three groups. *p* < 0.05 was considered to be statistically significant.

## Results

### Characterisation of sEVs derived from hAMSCs

**Fig. 1:**
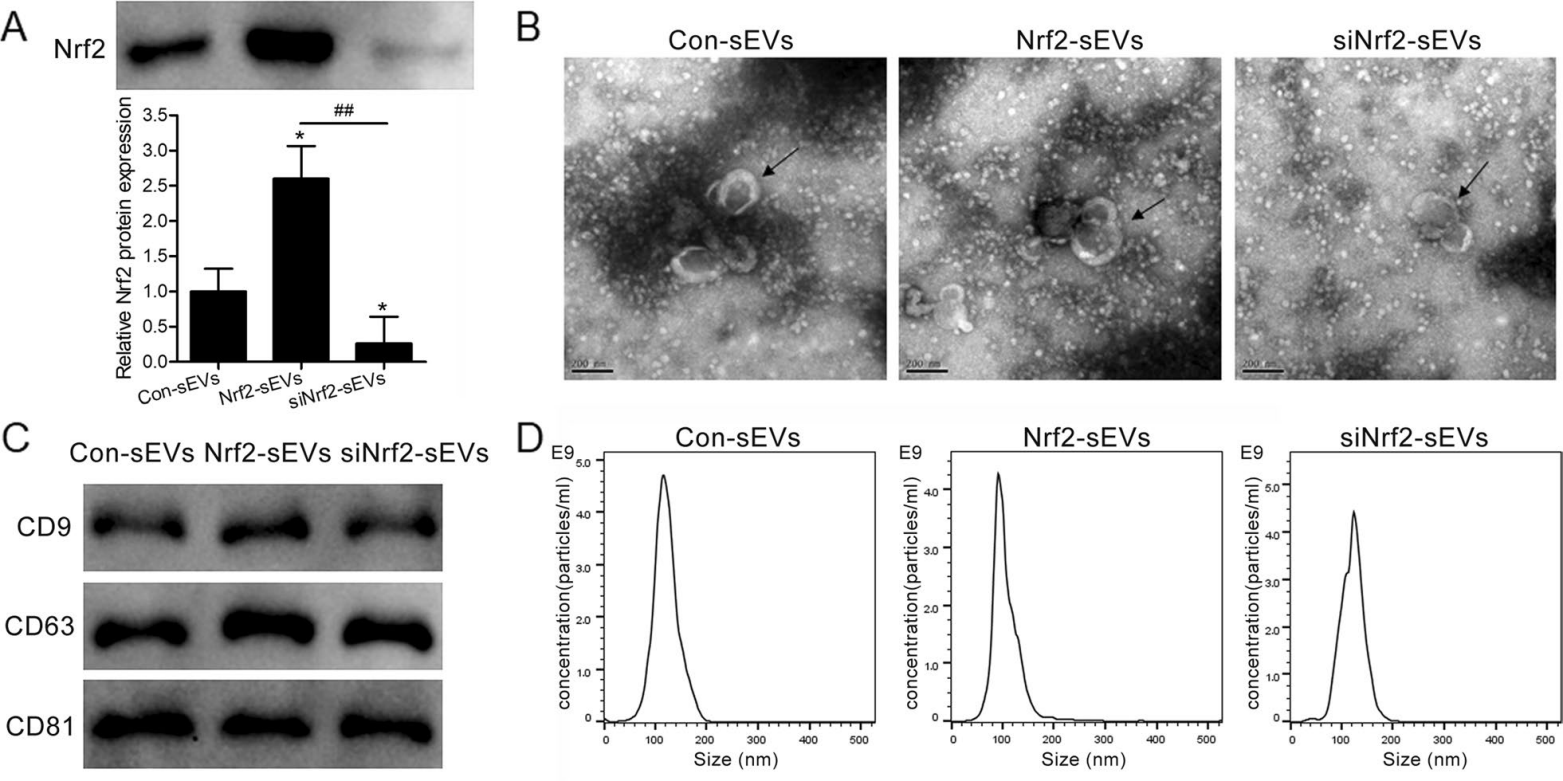
Characterisation of sEVs derived from hAMSCs. sEVs were purified from hAMSCs. Con-sEVs: sEVs isolated from hAMSCs; Nrf2-sEVs: sEVs isolated from hAMSCs overexpressing Nrf2. siNrf2-sEVs: sEVs isolated from hAMSCs with Nrf2 knockdown. **A** Nrf2 expression levels in Con-sEVs, Nrf2-sEVs and siNrf2-sEVs were analyzed by western blot. The data are presented as the mean ± SD of 3 samples per group. *p < 0.05 versus Con-sEVs, and ^##^*p* < 0.01 vs. Nrf2-sEVs. **B** Representative transmission electron microscopy images of Con-sEVs, Nrf2-sEVs and siNrf2-sEVs. The black arrows indicate sEVs. Scale bar = 200 nm. **C** Expression of sEVs markers (CD9, CD63, CD81) was analyzed by western blot. **D** Nanoparticle tracking analysis (NTA) was used to analyze the sEVs size and concentration

We have recently shown that hAMSCs over-expressing Nrf2 are potent inhibitors of LPS-induced lung injury [19]. We next examined whether sEVs isolated from Nrf2-expressing hAMSCs could also provide protection against ALI. First, we purified sEVs from hAMSCs (Con-sEVs), hAMSCs overexpressing Nrf2 (Nrf2-sEVs) or hAMSCs with knockdown of Nrf2 (siNrf2-sEVs) and confirmed that the Nrf2 overexpression and knockdown sEVs increased and decreased Nrf2 protein expression levels, respectively (Fig. 1A). In addition, we found that overexpression or knockdown of Nrf2 did not affect sEVs structure (Fig. 1B), expression of sEVs markers such as CD9, CD63 and CD81 (Fig. 1C) or sEVs size (Fig. 1D).

### Nrf2-sEVs protect against ALI

**Fig. 2:**
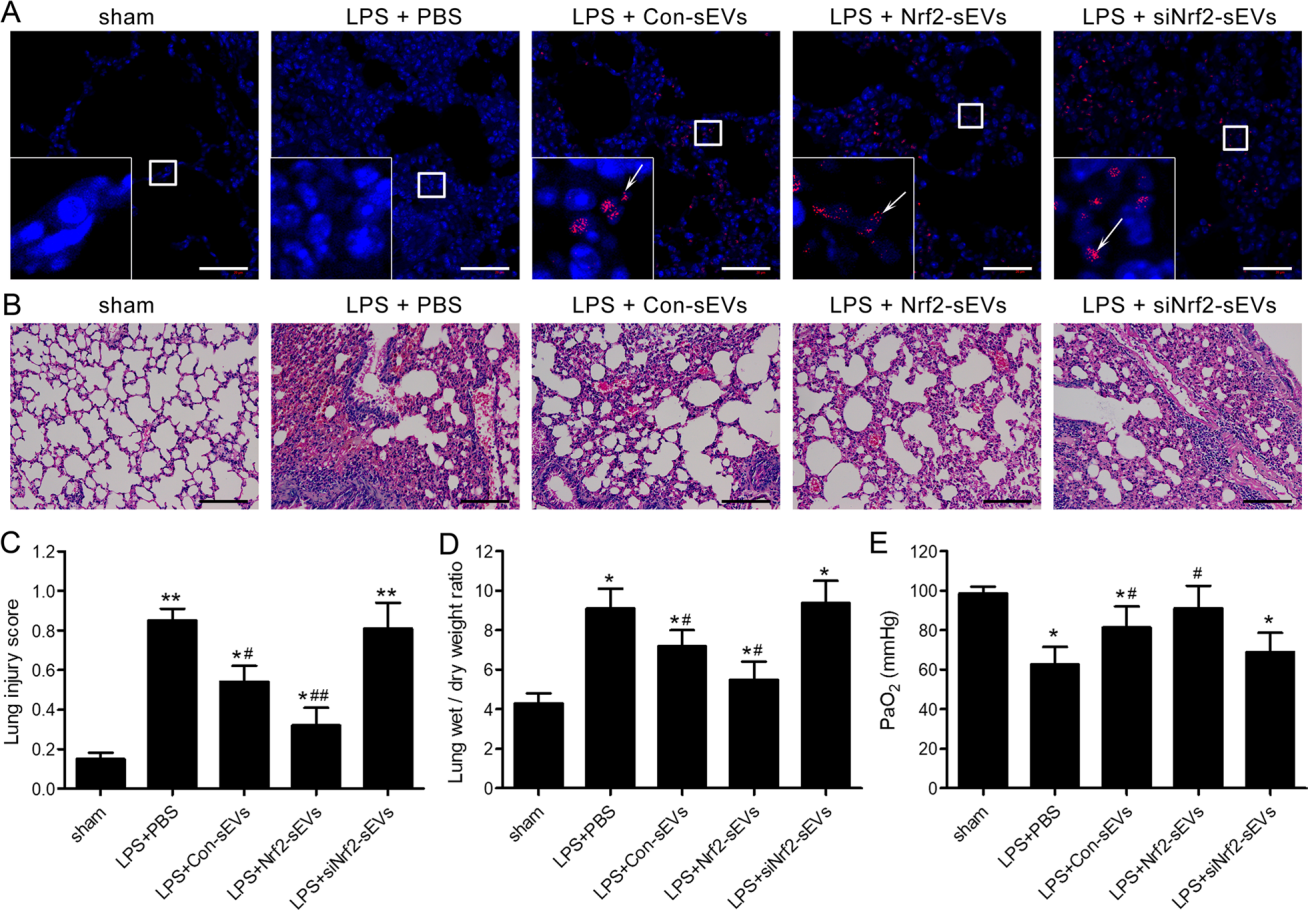
Nrf2-sEVs protect against ALI.ALI was induced by intratracheal instillation of LPS (100 µg in 50 µl saline). sEVs (or PBS control) were given intravenously at 2 and 12 h post-LPS administration. BALF and lung tissues were harvested at 24 h post-LPS. **A** Representative immunofluorescence images of PKH26-labeled sEVs in the lung tissue (arrows indicate sEVs). Scale bar = 50 μm. **B** Representative images of pathological changes in the lung tissue detected by H&E staining. Scale bar = 100 μm. **C** Lung injury was assessed using the histologic lung injury scoring system. **D** The wet/dry weight ratio of lung tissues. **E** Arterial oxygen tension (PaO_2_) was detected. n = 5 mice per group. Data are presented as mean ± SD. *p < 0.05, **p < 0.01 versus sham, and ^#^*p* < 0.05, ^##^*p* < 0.01 versus LPS + PBS group

Next, we sought to determine whether hAMSC-derived sEVs could protect against ALI in a mouse model. Following transfer, PKH26-labeled sEVs were observed after LPS induction in Con-sEVs, Nrf2-sEVs and siNrf2-sEVs groups but not in the sham or LPS + PBS groups (Fig. 2A). The pathological lung damage caused by LPS treatment was detected by H&E staining (Fig. 2B) and quantified using the histological lung injury scoring system (Fig. 2C). Treatment of mice with Con-sEVs provided significant protection against ALI (Fig. 2B, C). Importantly, this protection was further enhanced after treatment with Nrf2-sEVs (Fig. 2B, C), while siNrf2-sEVs were ineffective at providing protection from disease (Fig. 2B, C). Furthermore, the wet/dry lung weight ratio was also significantly decreased after treatment with Nrf2-sEVs compared to the PBS treatment group (Fig. 2D). In addition, Nrf2-sEVs treatment inhibited the LPS-induced decrease in arterial blood PaO2 (Fig. 2E). Taken together, these results suggest that sEVs derived from hAMSCs are protective, and that overexpression of Nrf2 in sEVs has a significant role in reducing lung injury.

### Nrf2-sEVs reduce apoptosis and inflammation in ALI

**Fig. 3:**
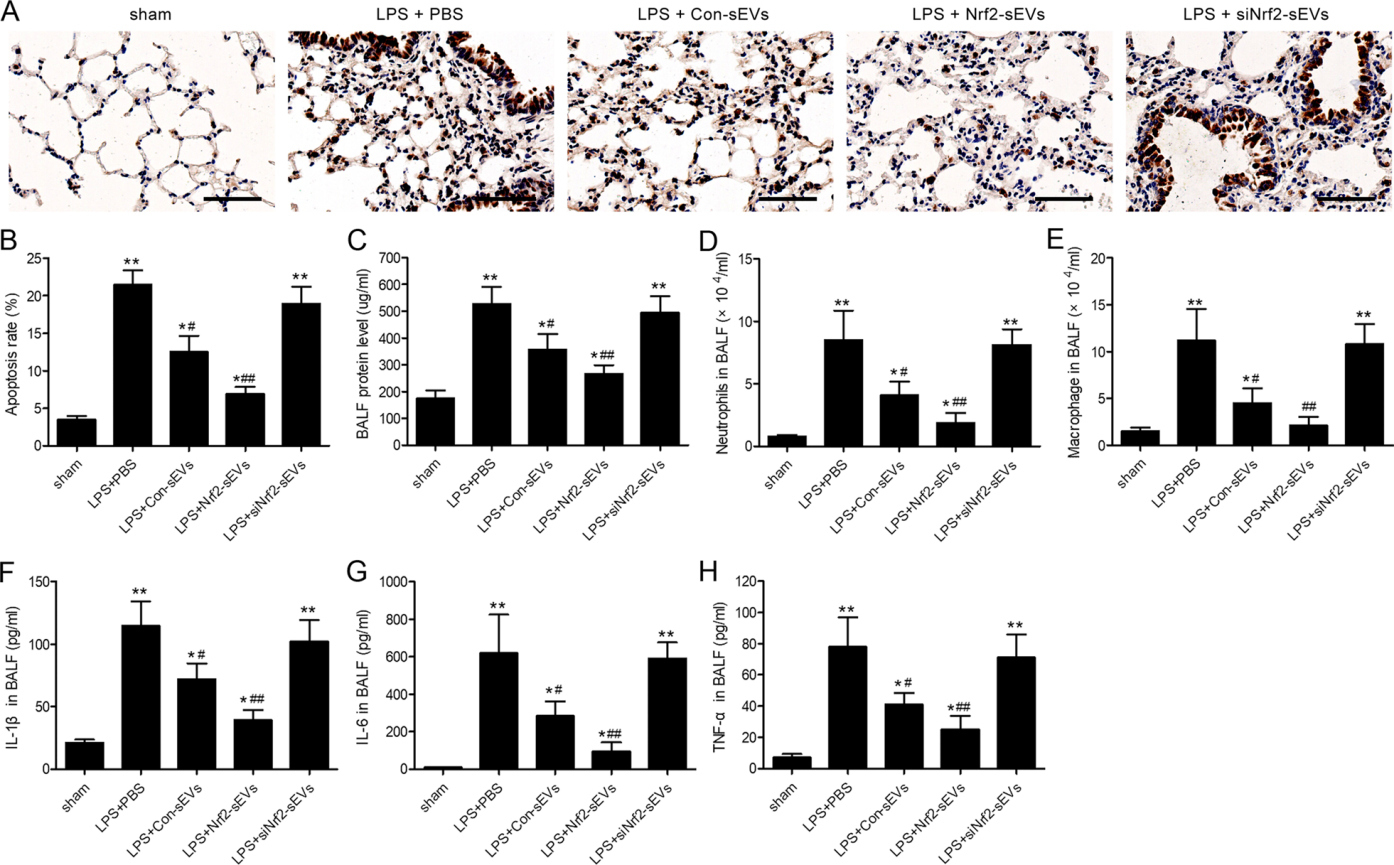
Nrf2-sEVs reduce apoptosis in ALI. (**A** and **B**) Apoptosis in lung tissue was evaluated by TUNEL staining and immunofluorescence microscopy. Scale bar = 100 μm. **C** The protein content of bronchoalveolar lavage fluid (BALF) was measured. (**D** and **E**) The number of neutrophil granulocytes (**D**) and macrophage (**E**) were counted in BALF at 24 h post-LPS stimulation. (**F**–**H**) ELISA was used to measure the protein expression levels of inflammatory cytokines, IL-1β (F), IL-6 (**G**) and TNF-α (**H**), in BALF at 24 h post-LPS stimulation. n = 5 mice per group. Data are presented as mean ± SD. *p < 0.05, **p < 0.01 versus sham, and ^#^*p* < 0.05, ^##^*p* < 0.01 versus LPS + PBS group

ALI is associated with inflammatory cell infiltration, production of inflammatory cytokines and epithelial cell apoptosis [48]. We used TUNEL staining to examine the level of apoptosis in each treatment group. We found that apoptosis was significantly reduced in Nrf2-sEVs compared to the PBS treatment group (Fig. 3A, B). Furthermore, treatment with Nrf2-sEVs also led to a significant reduction in the LPS-induced increase in BALF protein levels (Fig. 3C), as well as neutrophil (Fig. 3D) and macrophage (Fig. 3E) numbers in mice BALF. Finally, protein expression levels of the pro-inflammatory cytokines, IL-1β, IL-6 and TNF-α were also significantly lower after treatment with Nrf2-sEVs compared to the PBS treatment group (Fig. 3F–H). Thus, our results demonstrate that Nrf2-sEVs reduce LPS-induced apoptosis and BALF protein levels, inhibit infiltration of neutrophils and macrophages, and decrease the production of pro-inflammatory cytokines.

### Nrf2-sEVs promote expression of the Nrf2/HO-1 pathway

**Fig. 4:**
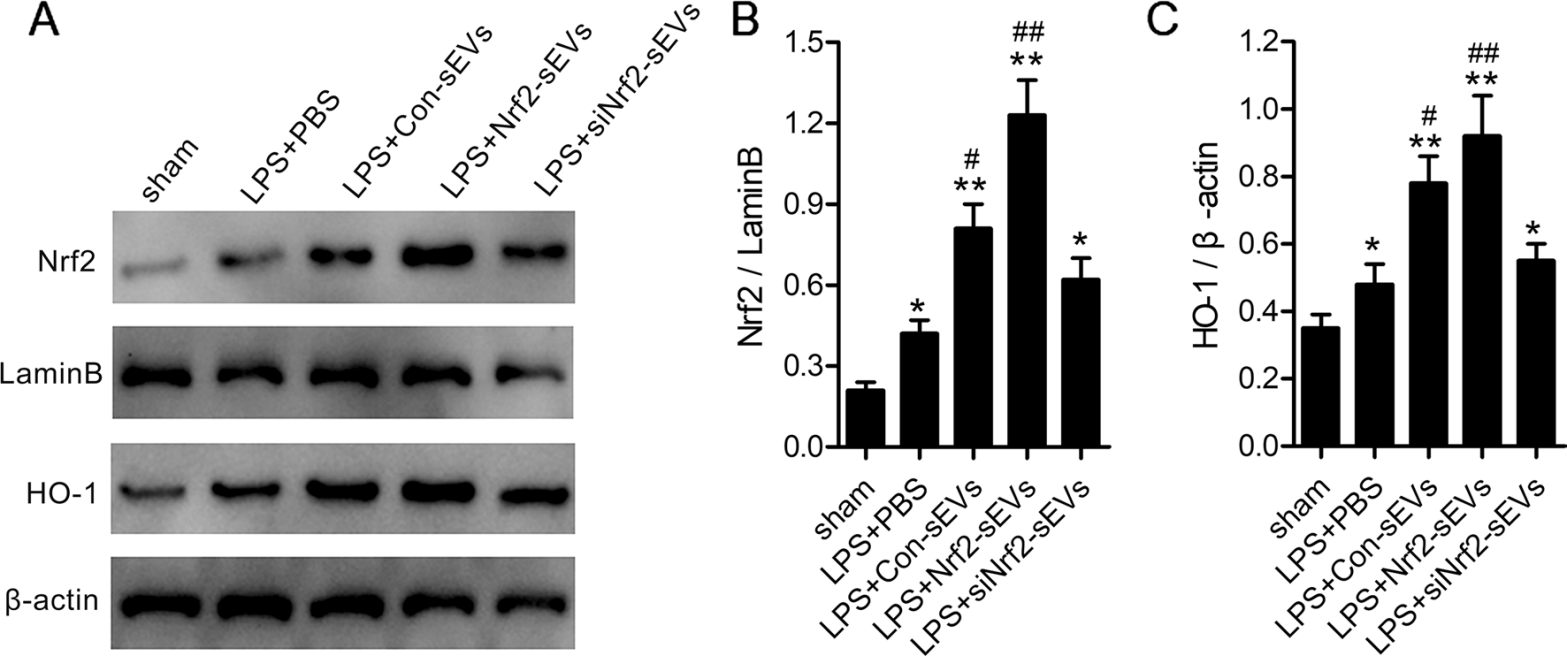
Nrf2-sEVs promote expression of the Nrf2/HO-1 pathway. **A** Western blot analysis was used to measure Nrf2 and HO-1 protein expression levels in lung tissue. **B** Nrf2 expression was normalized using LaminB levels. **C** HO-1 expression was normalized using β-actin levels. n = 5 mice per group. Data are presented as mean ± SD. *p < 0.05, **p < 0.01 versus sham, and ^#^*p* < 0.05, ^##^*p* < 0.01 versus LPS + PBS group

Since Nrf2 has been shown to be involved in mediating an antioxidant response via the transcription and synthesis of the antioxidant enzyme heme oxygenase-1 (HO-1) [49, 50], we next examined whether Nrf2-sEVs activated the Nrf2/HO-1 pathway. We found that both Nrf2 and HO-1 protein expression levels were significantly higher after treatment of LPS-injured mice with Nrf2-sEVs compared to the PBS, Con-sEVs and siNrf2-sEVs groups (Fig. 4A–C) in the lung tissue suggesting that sEVs expressing Nrf2 is functional and can transcriptionally activate expression of HO-1 during LPS-induced lung damage.

### Nrf2-sEVs inhibit activation of the NLRP3 inflammasome in LPS-damaged lung tissue

**Fig. 5:**
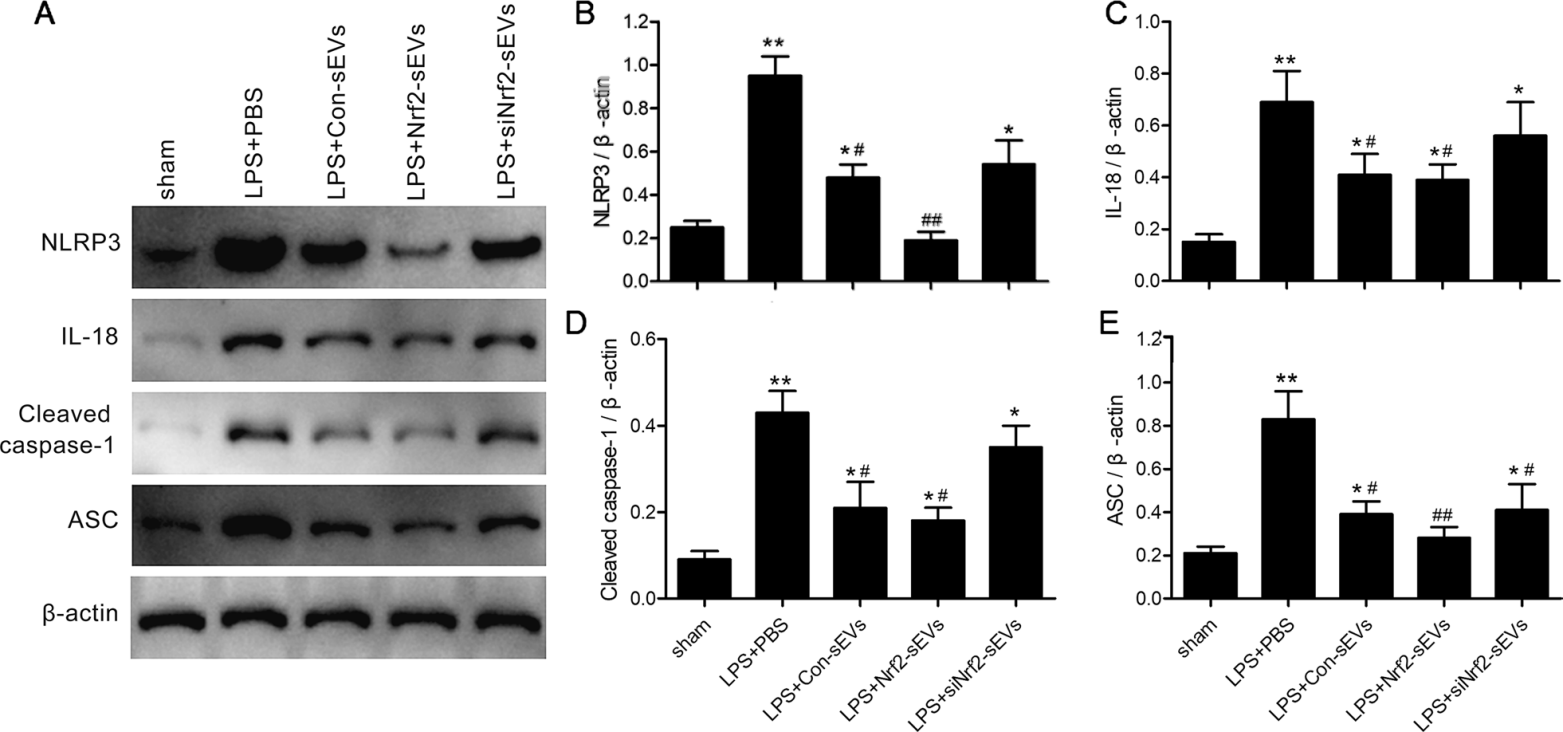
Nrf2-sEVs inhibit activation of the NLRP3 inflammasome. **A** Western blot analysis was used to examine NLRP3, IL-18, cleaved caspase-1 and ASC protein expression levels. **B**–**E** Protein expression was quantified using β-actin. n = 5 mice per group. Data are presented as mean ± SD. *p < 0.05, **p < 0.01 versus sham, and ^#^*p* < 0.05, ^##^*p* < 0.01 versus LPS + PBS group

**Fig. 6:**
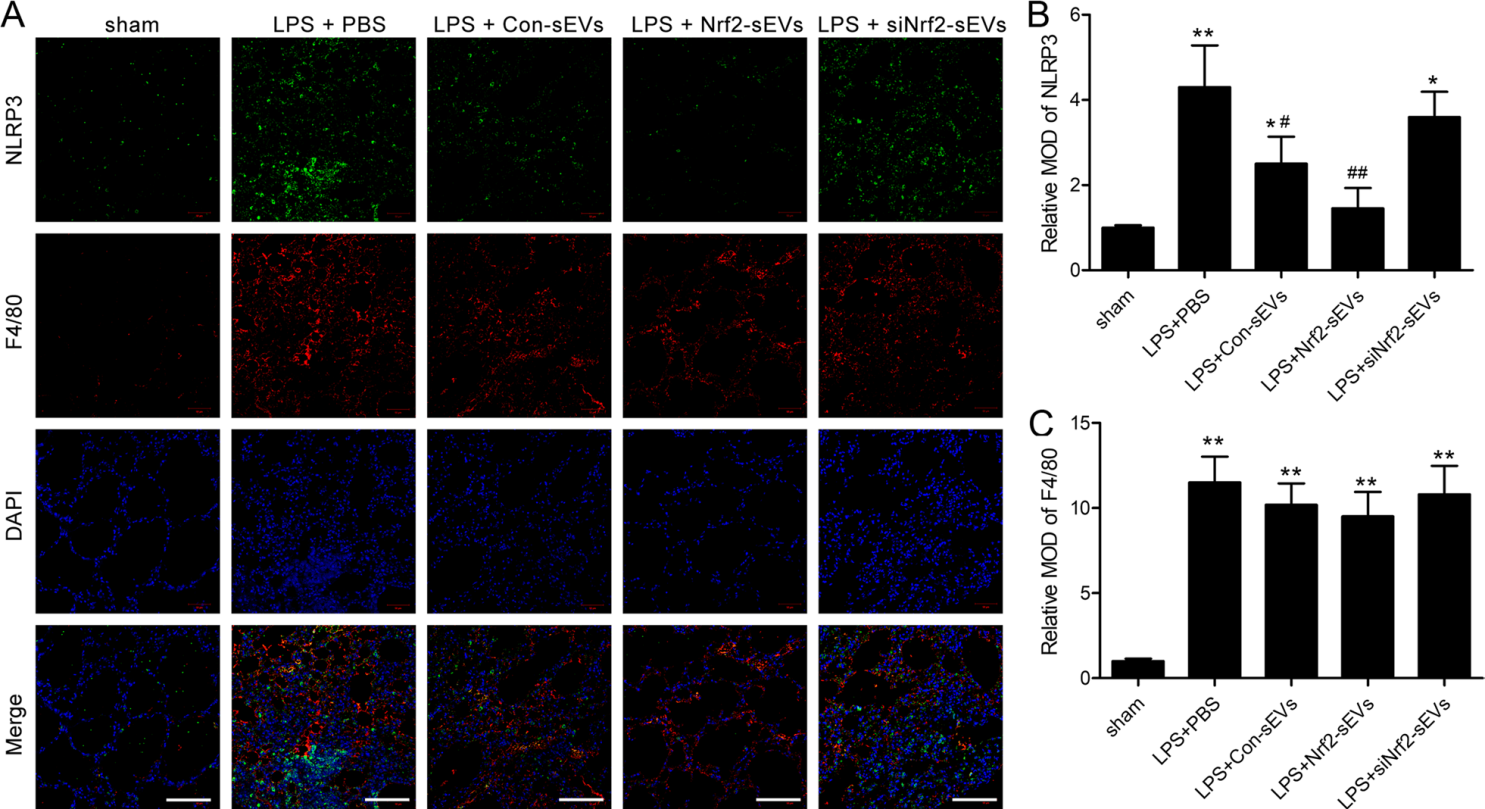
Nrf2-sEVs inhibit NLRP3 expression in macrophages. **A** Representative immunofluorescence images of lung tissue showing double-labeling of NLRP3 (green) and the murine macrophage marker, F4/80 (red). Scale bar = 100 μm. **B**, **C** Quantification of NLRP3 (**B**) and F4/80 (**C**) levels using the mean optical density (MOD). n = 5 mice per group. Data are presented as mean ± SD. *p < 0.05, **p < 0.01 versus sham, and ^#^*p* < 0.05, ^##^*p* < 0.01 versus LPS + PBS group

To determine whether NLRP3 is involved in regulating the Nrf2-mediated reduction in LPS-induced lung damage, we next examined the relationship between Nrf2 expression and NLRP3 levels. We found that treatment with Nrf2-sEVs led to a significant reduction in NLRP3 protein expression in the lung tissue compared to the PBS, Con-sEVs and siNrf2-sEVs groups (Fig. 5A, B). Similarly, downstream markers of inflammasome activation including IL-18 (Fig. 5C), cleaved Caspase-1 (Fig. 5D) and ASC (Fig. 5E) were significantly decreased after treatment with Nrf2-sEVs. Immunofluorescence staining was used to examine the expression patterns of NLRP3 in LPS-damaged lung tissue. We found that NLRP3 levels were significantly increased after LPS induction and that treatment with Nrf2-sEVs significantly reduced NLRP3 expression (Fig. 6A, B). We further show that treatment with sEVs had no effect on macrophage levels (measured by F4/80 expression) in the lung tissue across all the LPS treatment groups (Fig. 6C). These results suggest that Nrf2-sEVs inhibit activation of the NLRP3 inflammasome during LPS-induced lung injury.

### Nrf2-sEVs promote M2 macrophage polarization

**Fig. 7:**
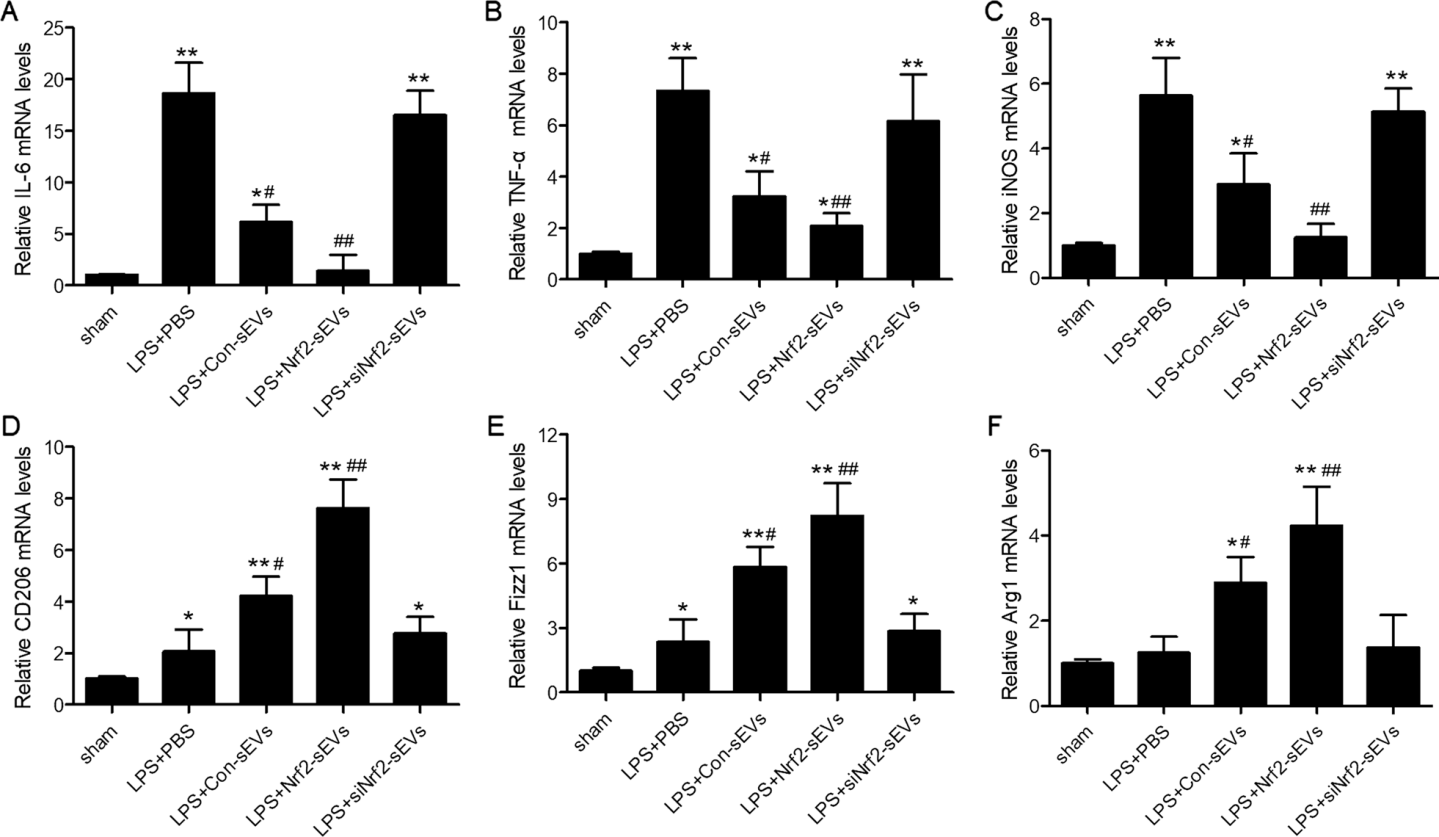
Nrf2-sEVs promote M2 macrophage differentiation. RT-PCR analysis was used to measure mRNA expression of M1-associated genes, IL-6 (**A**), TNF-α (**B**) and iNOS (**C**), and M2-associated genes, CD206 (**D**), Fizz1 (**E**) and Arg1 (**F**) in lung tissues. n = 5 mice per group. Data are presented as mean ± SD. *p < 0.05, **p < 0.01 versus sham, and ^#^*p* < 0.05, ^##^*p* < 0.01 versus LPS + PBS group

**Fig. 8:**
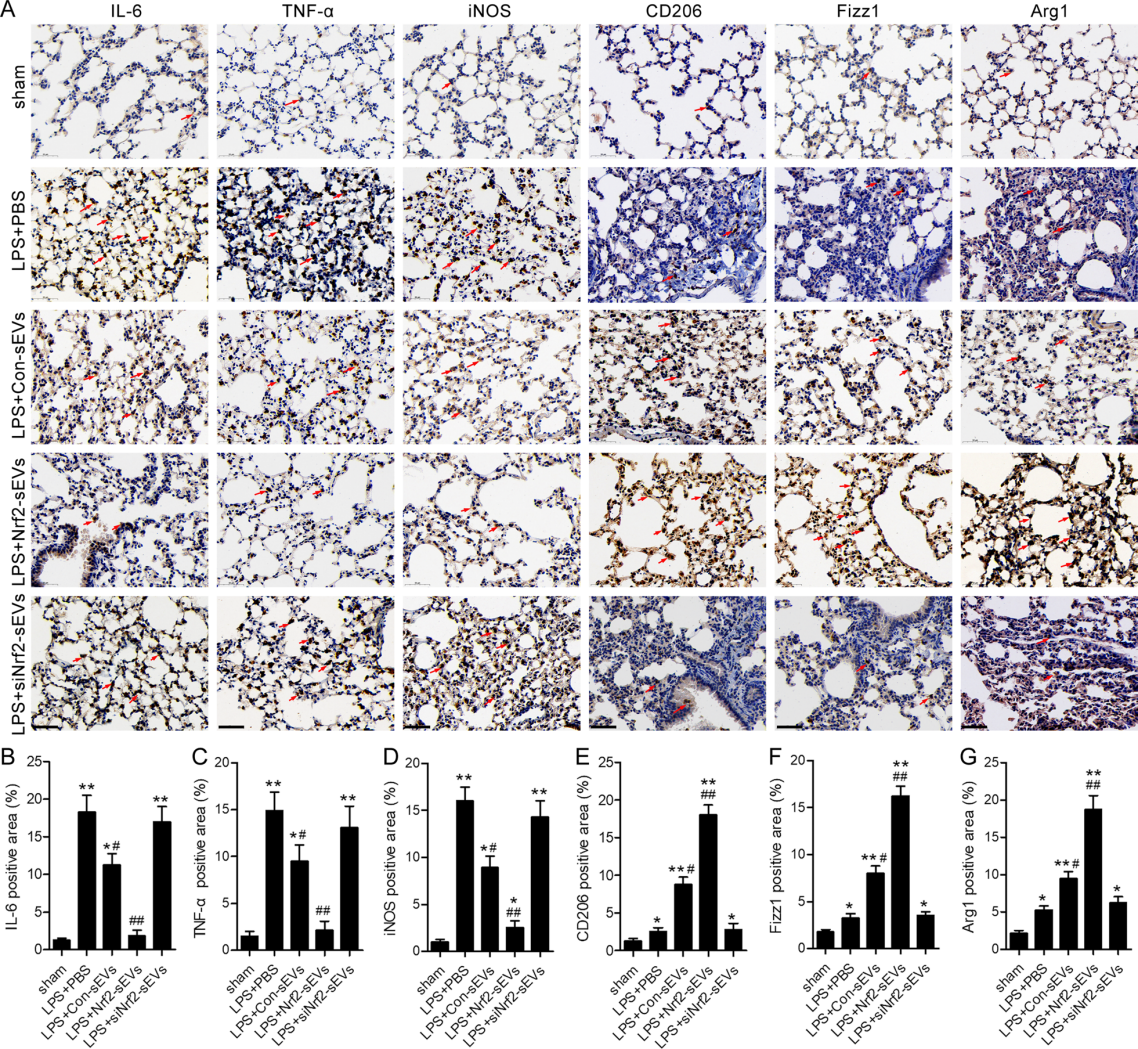
Immunohistochemical analysis was used to measure protein expression of M1- and M2-associated genes (**A**). Red arrows indicated positive staining. Scale bar = 50 μm. Quantification of percentage positive IL-6 (**B**), TNF-α (**C**) and iNOS (**D**), CD206 (**E**), Fizz1 (**F**) and Arg1 (**G**) staining area. n = 5 mice per group. Data are presented as mean ± SD. *p < 0.05, **p < 0.01 versus sham, and ^#^*p* < 0.05, ^##^*p* < 0.01 versus LPS + PBS group

Since we observed high levels of lung tissue macrophages in all groups following LPS-induced lung damage, we next examined whether distinct macrophage subsets were present in the lung tissue. A previous study has shown that Nrf2 activation promotes M2 macrophage differentiation [37]. qRT-PCR and immunohistochemical analyses were used to quantify levels of various M1 (IL-6, TNF-α and relative iNOS) and M2 (CD206, Fizz1 and Arg1) macrophage markers. We found that in LPS-injured mice treated with Nrf2-sEVs, there was a significant decrease in M1 macrophage markers and a significant increase in M2 macrophage markers compared to PBS, Con-sEVs and siNrf2-sEVs groups (Figs. 7 and 8). These results suggest that there is a shift from M1 to M2 macrophages, which is consistent with the protective effects of Nrf2-sEVs on LPS-induced lung injury.

## Discussion

In this study, we demonstrate that sEVs derived from hAMSCs overexpressing Nrf2 (Nrf2-sEVs) protect against the development of LPS-induced lung injury. Treatment with Nrf2-sEVs led to significant amelioration of lung inflammation, which was associated with reduced activation of the NLRP3 inflammasome and increased differentiation of M2 macrophages.

ALI remains a significant problem in critical care [51], especially since current therapies are limited primarily to respiratory support and immunosuppression [7]. The advent of cellular therapies to treat inflammatory diseases is providing new therapeutic options for these patients [3]. MSCs have emerged as important modulators of immune-mediated inflammation and exploiting their potential may lead to future therapies [8]. We previously showed that MSCs derived from human amniotic membranes provided protection in a mouse model of lung injury [19]. However, MSC-based therapies are complex due to the isolation, purification and sterile storage of MSCs, and inflammatory microenvironments are not conducive to the survival of transplanted MSCs, so the use of sEVs may be an alternative approach to mimic MSC treatments [20]. MSC-derived sEVs have been shown to promote protection in a variety of inflammatory diseases [21]. A recent study has shown that hAMSC-derived sEVs promote cutaneous wound healing in a mouse model [52]. Here, we show for the first time that sEVs from hAMSCs protect against LPS-induced lung inflammation. Thus, hAMSC-derived sEVs may be a viable therapeutic strategy to treat inflammatory diseases.

Nrf2 is a transcription factor that controls antioxidant gene expression [32]. Nrf2 has also been shown to regulate the development of lung injury by inducing antioxidant genes, modulating the NLRP3 inflammasome and promoting M2 macrophage polarization [37]. We previously showed that overexpression of Nrf2 in hAMSCs enhanced the protective effects of hAMSCs alone [19]. Consistent with these findings, we now show that sEVs from Nrf2-overexpressing hAMSCs also provide increased protection against lung injury, while the beneficial effect was reversed by sEVs from siNrf2-hAMSCs. Thus, we identify Nrf2 as an important regulatory factor in limiting lung inflammation.

Nrf2 has been shown to inhibit assembly and activation of the NLRP3 inflammasome [32, 39]. We also find that treatment of mice with Nrf2-sEVs following LPS challenge results in a significant reduction in expression of NLRP3 and its downstream effectors, cleaved Caspase-1, ASC and IL-18. These findings indicate that the Nrf2/NLRP3 pathway could be exploited as a therapeutic target in ALI.

Macrophages have the ability to acquire distinct phenotypes and functions in response to external stimuli. M1 macrophages develop in the presence of inflammatory mediators such as LPS and TNFα, while M2 macrophages are dependent upon signals such as IL-4 or IL-13 [53]. M1 macrophages produce pro-inflammatory cytokines such as IL-12 and promote inflammation, while M2 macrophages are associated with wound healing and tissue repair. Here, we find that treatment with Nrf2-sEVs promotes expression of M2 macrophage markers and reduces expression of M1 markers, suggesting a switch in the polarization of macrophages from an inflammatory phenotype to a healing role.

Taken together, we show that sEVs derived from Nrf2-overexpressing hAMSCs protect against LPS-induced lung injury by inhibiting the activation of the NLRP3 inflammasome and promoting the polarization of M2 macrophages. These findings suggest that Nrf2 overexpression may protect against lung inflammation and provide a novel therapeutic approach for ALI. Future studies in other models of lung inflammation may provide the rationale for examining the efficacy of Nrf2-sEVs in human patients. However, our study does have some limitations. Firstly, although Nrf2-sEVs significantly reduced the levels of macrophages in BALF, macrophages levels in lung tissue decreased only slightly, perhaps due to delayed expression in lung tissue [54]. It is therefore necessary for future studies to monitor the macrophage levels in lung tissue over different time periods. Secondly, we speculate that the reduced expression of IL6 and TNFα is due to distinct macrophage subsets in the lung tissue. Meanwhile, studies have shown that Nrf2 activation increased the phagocytic ability of THP-1 macrophages [55], and inhibition of NRF2 signaling suppresses the phagocytosis of *P. aeruginosa* by RAW264.7 macrophages [56]. Therefore, we speculate also that Nrf2-Exo may promote M2 macrophage phagocytosis. However, in the absence of analyzing isolated macrophages from the lung tissue, further in vitro studies in macrophage cell lines are required to confirm that this is the case. Finally, NLRP3 expression partly overlapped with F4/80 in macrophages, it remains unclear which other cell types are involved in responding to LPS stimulation with activation of NLRP3 inflammasome.

## Data Availability

All data generated or analyzed in this study are included in this article, and are available from the corresponding author on request.
